# 胸腔镜与开放手术治疗临床早期胸腺恶性肿瘤的围手术期效果及长期生存率的比较

**DOI:** 10.3779/j.issn.1009-3419.2016.07.07

**Published:** 2016-07-20

**Authors:** 灏 汪, 志涛 谷, 建勇 丁, 黎杰 谭, 剑华 傅, 毅 沈, 煜程 魏, 鹏 张, 泳涛 韩, 椿 陈, 仁泉 张, 印 李, 克能 陈, 和忠 陈, 永煜 刘, 有斌 崔, 允 王, 烈文 庞, 振涛 于, 鑫明 周, 阳春 柳, 媛 刘, 文涛 方, 中国胸腺肿瘤协作组成员

**Affiliations:** 1 200032 上海，复旦大学附属中山医院胸外科 Department of Thoracic Surgery, Zhongshan Hospital, Fudan University, Shanghai 200032, China; 2 200030 上海，上海交通大学附属上海胸科医院 Department of Thoracic Surgery, Shanghai Chest Hospital, Shanghai Jiao Tong University, Shanghai 200030, China; 3 510060 广州，中山大学附属肿瘤医院胸外科 Department of Thoracic Surgery, Guang dong Esophageal Cancer Institute, Sun Yat-sen University Cancer Center, State Key Laboratory of Oncology in South China, Collaborative Innovation Center of Cancer Medicine, Guangzhou 510060, China; 4 266001 青岛大学医学院附属医院胸外科 Department of Thoracic Surgery, Afliated Hospital of Qingdao University, Qingdao 266001, China; 5 300052 天津，天津医科大学附属总医院胸外科 Department of Endocrinology, Tianjin Medical University General Hospital, Tianjin 300052, China; 6 610041 成都，四川省肿瘤医院胸外科 Department of Thoracic Surgery, Sichuan Cancer Hospital, Chengdu 610041, China; 7 350001 福州，福建医科大学附属协和医院胸外科 Department of Thoracic Surgery, Fujian Medical University Union Hospital, Fuzhou 350001, China; 8 230022 合肥，安徽医科大学附属第一医院胸外科 Department of Thoracic Surgery, First Afliated Hospital of Anhui Medical University, Hefei 230022, China; 9 450008 郑州，郑州大学附属肿瘤医院胸外科 Department of Thoracic Surgery, Afliated Cancer Hospital of Zhengzhou University, Zhengzhou 450008, China; 10 100142 北京，北京大学附肿瘤医院胸外科 Department of Thoracic Surgery, Beijing Cancer Hospital, Beijing 100142, China; 11 200433 上海，长海医院胸心外科 Department of Cardiothoracic Surgery, Changhai Hospital, Shanghai 200433, China; 12 110042 沈阳，辽宁肿瘤医院胸外科 Department of Thoracic Surgery, Liaoning Cancer Hospital, Shenyang 110042, China; 13 130021 长春，吉林大学附属第一医院胸外科 Department of Thoracic Surgery, First Afliated Hospital of Jilin University, Changchun 130021, China; 14 610041 成都，四川大学华西医院胸外科 Department of Thoracic Surgery, West China Hospital, Sichuan University, Chengdu 610041, China; 15 200032 上海，复旦大学附属华山医院胸外科 Department of Thoracic Surgery, Huashan Hospital, Fudan University, Shanghai 200032, China; 16 300060 天津，天津医科大学附属肿瘤医院食管癌中心 Department of Esophageal Cancer, Tianjin Cancer Hospital, Tianjin 300060, China; 17 310022 杭州，浙江省肿瘤医院胸外科 Department of Thoracic Surgery, Zhejiang Cancer Hospital, Hangzhou 310022, China; 18 330006 南昌，江西省人民医院胸外科 Department of Thoracic Surgery, Jiangxi People's Hospital, Nanchang 330006, China

**Keywords:** 胸腺恶性肿瘤, 胸腺切除术, 胸腔外科, 开胸手术, Thymic malignancies, Tymectomy, Video-assisted Thoracoscopic Surgery (VATS), Open surgery

## Abstract

**背景与目的:**

胸腔镜胸腺切除术相比开放手术治疗早期（Masaoka-Koga Ⅰ期或Ⅱ期）胸腺恶性肿瘤在理论上具有优势，然而尚未有研究报道其在长期生存率方面的差异。本研究基于中国胸腺肿瘤研究协作组（Chinese Alliance for Research in Thymomas, ChART）的数据库对此进行了研究。

**方法:**

以数据库中1994年到2012年间的1, 117例早期（Masaoka-Koga Ⅰ期或Ⅱ期）胸腺恶性肿瘤患者为研究对象。其中241例行胸腔镜胸腺切除术，876例行开放手术。采用单因素分析比较两组的临床资料与围手术期结果方面的差异。采用多因素分析明确影响长期预后的相关因素。

**结果:**

与开放手术组相比，胸腔镜组的全胸腺切除比例更高（80.5% *vs* 73.9%, *P*=0.028），根治性切除率更高（98.8% *vs* 88.7%, *P* < 0.000），而复发率更低（2.9% *vs* 16.0%, *P* < 0.001），5年无瘤生存率更高（92% *vs* 83%, *P*=0.011），而两组的5年生存率接近（92% *vs* 92%, *P*=0.15）。*Cox*比例风险模型分析显示WHO分型、Masaoka-Koga分期和术后辅助治疗是影响胸腺恶性肿瘤长期生存的独立因素。

**结论:**

胸腔镜胸腺切除术是治疗早期胸腺恶性肿瘤安全有效的方法，与开放手术相比其围手术期效果更好，肿瘤学疗效一致。

## Abstract (en)

**Background and objective:**

Video-assisted thoracoscopic surgery (VATS) theoretically offers advantages over open thymectomy for clinically early-stage (Masaoka-Koga stage Ⅰ and Ⅱ) thymic malignancies. However, longterm outcomes have not been well studied. We compared the postoperative outcomes and survival from a cohort study based on the database of the Chinese Alliance for Research in Thymomas (ChART).

**Methods:**

Between 1994 and 2012, data of 1, 117 patients having surgery for clinically early-stage (Masaoka-Koga stage Ⅰ and Ⅱ) tumors were enrolled for the study. Among them, 241 cases underwent VATS thymectomy (VATS group), while 876 cases underwent open thymectomy (Open group). Univariate analyses were used to compare the clinical character and perioperative outcomes between the two groups. And multivariate analysis was performed to determine the independent predictive factors for long-term survival.

**Results:**

Compared with the Open group, the VATS group had higher percentage of total thymectomy (80.5% *vs* 73.9%, *P*=0.028), resection rate (98.8% *vs* 88.7%, *P* < 0.001) and less recurrence (2.9% *vs* 16.0%, *P* < 0.001). Five-year overall survival was 92% afer VATS and 92% afer open thymectomy, with no signifcant difference between the two groups (*P*=0.15). However, 5-year disease free survival were 92% in VATS group and 83% in Open group (*P*=0.011). *Cox* proportional hazards model revealed that WHO classifcation, Masaoka-Koga stage and adjuvant therapy were independent predictive factors for overall survival, while surgical approach had no signifcant impact on long-term outcome.

**Conclusion:**

Tis study suggests that VATS thymectomy is an effective approach for clinically early-stage thymic malignancies. And it may offer better perioperative outcomes, as well as equal oncological survival.

微创手术在胸腺肿瘤的治疗中得到了越来越广的应用。近年来的几项研究^[1-4]^提示，胸腔镜手术相比开放手术的短期临床效果更好。然而这些研究样本容量较小，尚不足以获得肯定性的结论。同时，手术方法对于胸腺恶性肿瘤长期疗效的差异的研究甚少，因为现有的报道并没有提供长期随访的结果。因此, 迄今为止的证据尚不足以明确胸腔镜手术相比开放手术孰优孰劣的结论^[5]^。本研究基于中国胸腺肿瘤研究协作组（Chinese Alliance for Research in Thymomas, ChART）的大样本数据库，对于两种手术方式治疗早期胸腺恶性肿瘤的围手术期的效果和长期生存率的情况进行了对比研究。

## 1 材料和方法

本研究对ChART数据库中所纳入的从1994至2012年间国内18家大中心的所有2, 370例胸腺恶性肿瘤患者进行了筛选。纳入标准是行手术治疗的临床早期（Masaoka-Koga Ⅰ期和Ⅱ期）患者。排除标准是术前经过了新辅助治疗的患者或者行保守治疗的患者。本研究符合赫尔辛基宣言的原则，并得到了所有医院的伦理委员会的批准。在这项多中心的回顾性研究中，手术方式的选择由各中心的手术医生根据患者的具体病情以及医师各自的经验进行决定。手术方式包括微创手术（胸腔镜）和开放手术（胸骨正中劈开手术、蚌壳式切口和侧胸切口）。使用SPSS 22.0软件进行统计学检验。连续变量的比较采用*t*检验；分类变量的比较采用卡方检验或者*Fisher*精确检验；采用*Kaplan-Meier*法计算生存曲线，采用*Log-rank*法比较生存率的差异；采用*Cox*比例风险模型进行多因素分析独立的预后因素。以双侧*P*值< 0.05作为差异具有统计学意义的标准。

## 2 结果

共有1, 117个病例纳入到本研究的分析之中。其中241例行胸腔镜手术，876例行开放手术。胸腔镜手术开始于2004年（图 1），之后所应用的比例越来越高，在近3年中已经超过了40%（图 2）。两组患者的临床基本资料见表 1。与开放组相比，胸腔镜组的女性比例和重症肌无力的比例更高。开放组的肿瘤直径更大，临床分期为进展期的比例更高、WHO分型的比例也更高。

**1:**
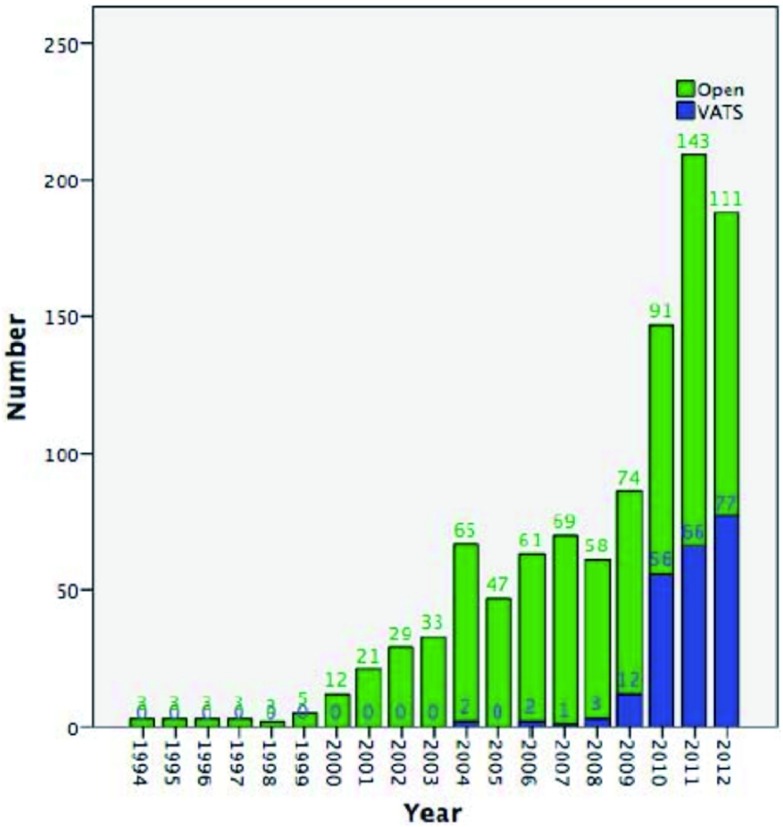
所有病例入组情况 The cases number of patients undergoing thymectomy, after open or VATS incision from 1994 to 2012

**2:**
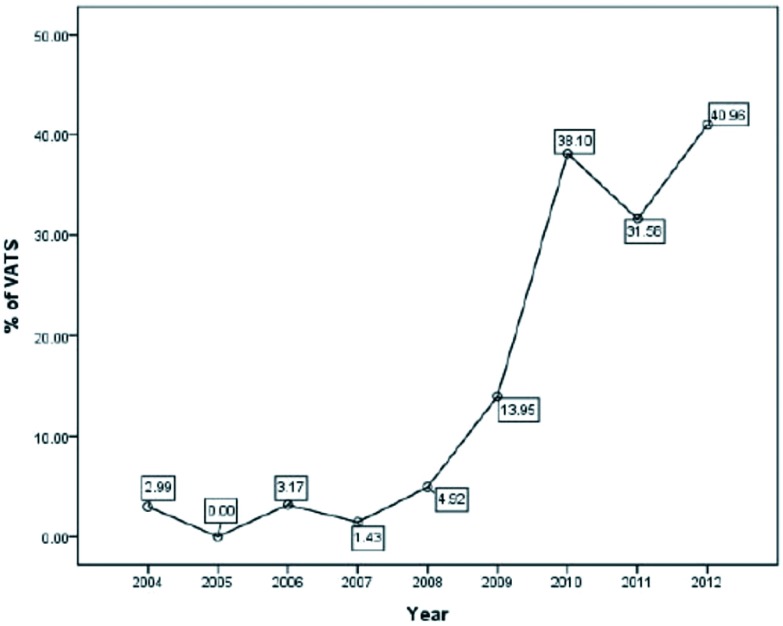
胸腔镜手术的比例变化 The percentage of patients after VATS incision from 2004 to 2012

**1:** 患者的临床资料 Patient demographics

Item	VATS group(*n*=241)	Open group(*n*=876)	*P*
Gender			0.027
Male	108(44.8%)	463(52.9%)	
Female	133(55.2%)	413(47.1%)	
Age(yr)	51.79	50.62	0.201
Myasthenia gravis			< 0.001
Yes	82(34.3%)	191(21.9%)	
No	157(65.7%)	682(78.1%)	
Clinical Stage			0.008
Ⅰ	183(75.9%)	587(67.0%)	
Ⅱ	58(24.1%)	289(33.0%)	
WHO classification			< 0.001
A+AB	100(41.5%)	282(32.2%)	
B1+B2+B3	127(52.7%)	406(46.3%)	
C	14(5.8%)	188(21.5%)	
Tumor size(cm)	4.65	7.17	< 0.001
Pathological stage			< 0.001
Ⅰ	168(71.5%)	386(44.2%)	
Ⅱ	61(26.0%)	224(25.6%)	
Ⅲ	3(1.3%)	213(24.4%)	
Ⅳ	3(1.3%)	51(5.8%)	
VATS: video-assisted thoracoscopic surgery; WHO: World Health Organization. 注：本表得到版权所有者©2011-2016 Journal of Thoracic Disease复制许可。			

在围手术期效果方面，胸腔镜组行全胸腺切除的比例更高（80.5% *vs* 73.9%, *P*=0.028），根治性切除比例更高（98.8% *vs* 88.7%, *P* < 0.001）。开放组有3例患者在术后30天内死亡, 而胸腔镜组无死亡（表 2）。

**2:** 围手术期指标 Perioperative outcomes

Item	VATS group(*n*=241)	Open group(*n*=876)	*P*
Extent of resection			0.028
Partial thymectomy	46(19.1%)	229(26.1%)	
Total thymectomy	195(80.5%)	647(73.9%)	
Resection status			< 0.001
R0	238(98.8%)	776(88.7%)	
R1	3(1.2%)	32(3.7%)	
R2	0(0.0%)	67(7.7%)	
30-day Mortality	0	3(0.34%)	1.000
VATS: video-assisted thoracoscopic surgery. 注：本表得到版权所有者©2011-2016 Journal of Thoracic Disease复制许可。			

在经过了中位数时间为33.5个月的随访期之后，两组的五年生存率相近（92% *vs* 92%, *P*=0.15)。胸腔镜组的复发率更低（2.9% *vs* 16.0%, *P* < 0.001），五年无瘤生存率更高（92% *vs* 83%, *P*=0.011）（图 3，图 4）。多因素分析显示，仅有WHO分型、Masaoka-Koga分期和术后辅助治疗是影响长期生存的独立因素（表 3），而手术方式对于长期生存而言不是一个独立的预后因素。

**3:**
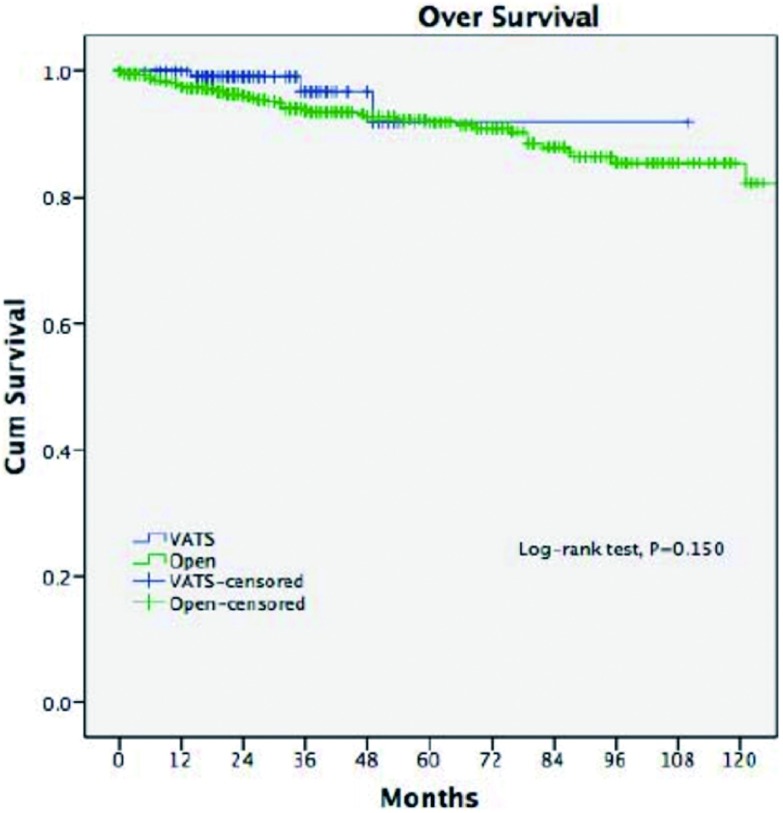
两组的总体生存率 Overall survival of two groups

**4:**
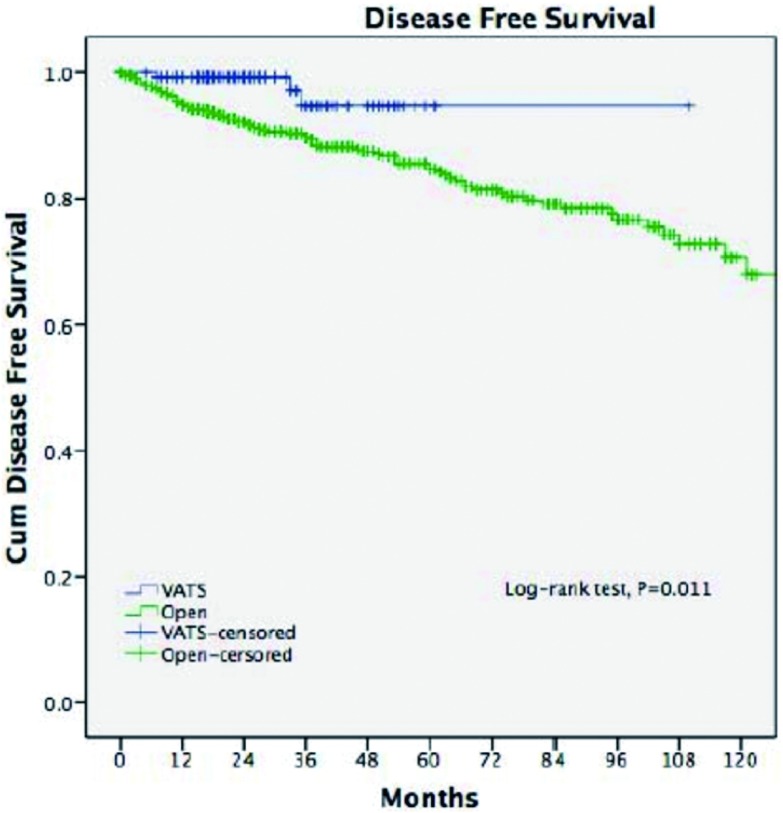
两组的无瘤生存率 Disease-free survival of two groups

**3:** 多因素分析(*Cox*比例风险模型) Multivariate analyses for survival(*Cox* proportional hazards model)

Factor	*P*	OR
Myasthenia gravis(No *vs* Yes)	0.307	0.617
WHO classification	0.001	
B1+B2+B3 *vs* A+AB	0.006	17.064
C *vs* B1+B2+B3	0.001	31.283
Masaoka stage	0.005	
Ⅱ *vs* Ⅰ	0.082	2.165
Ⅲ *vs* Ⅱ	0.002	3.421
Ⅳ *vs* Ⅲ	0.001	5.886
Adjuvant therapy(Yes *vs* No)	0.010	2.984
Surgical Approach(VATS *vs* Open)	0.374	1.956
Tumor Size(≤5 cm *vs* >5 cm)	0.721	1.124
Resection status(R1+R2 *vs* R0)	0.397	0.767
VATS: video-assisted thoracoscopic surgery; WHO:World Health Organization. 注：本表得到版权所有者©2011-2016 Journal of Thoracic Disease复制许可。		

鉴于两组患者在临床资料和病理分期上的不一致性，我们挑选了Masaoka-Koga分期为Ⅰ期和Ⅱ期的早期病例进行进一步的分析，包括胸腔镜组229例和开放组610例。两组患者在五年总体生存率（89.4% *vs* 96.7%, *P*=0.582）和复发率（3.3% *vs* 4.7%, *P*=0.579）方面均无统计学差异（图 5，图 6）。

**5:**
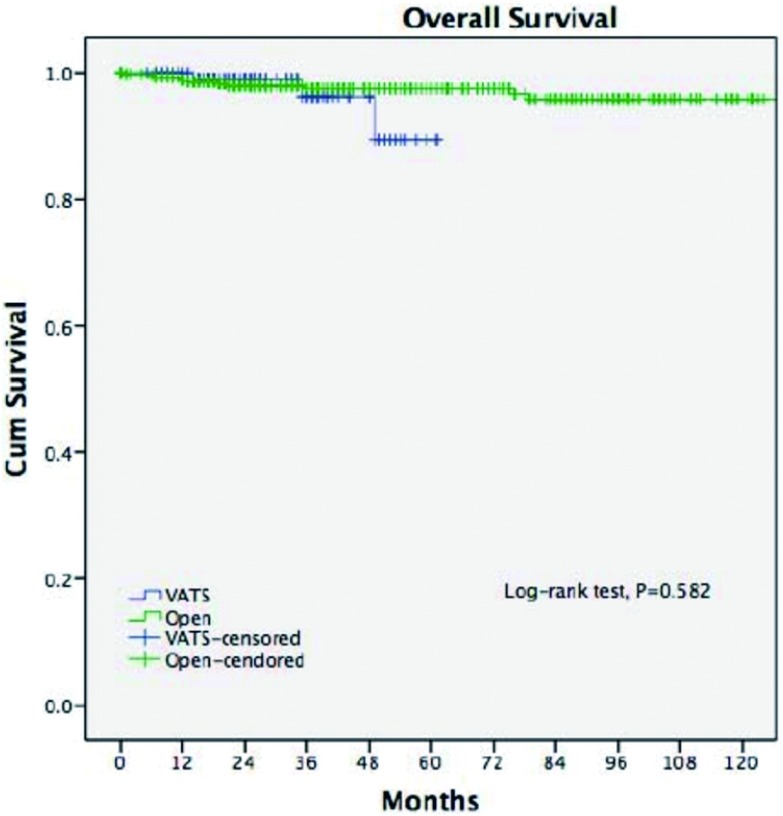
术后病理分期为早期（Masaoka-Koga Ⅰ和Ⅱ期）的胸腔镜组与开放组患者的五年生存率(89.4% *vs* 96.7%, *P*=0.582) For Masaoka-Koga pStage Ⅰ-Ⅱ tumors, the 5-year overall survival (89.4% *vs* 96.7%, *P*=0.582) was similar between the two groups

**6:**
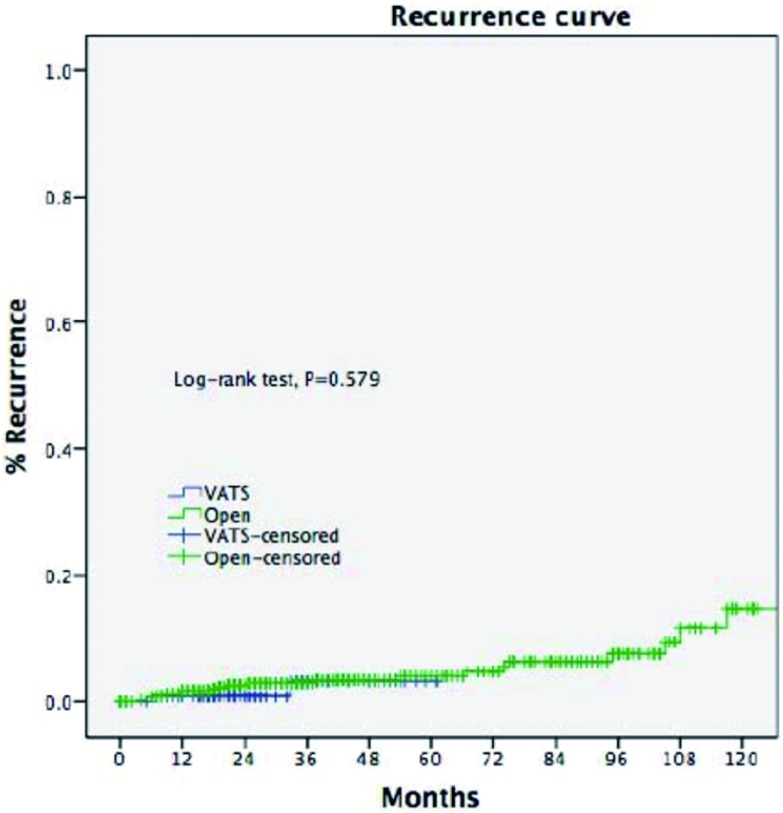
术后病理分期为早期（Masaoka-KogaⅠ和Ⅱ期）的胸腔镜组与开放组患者的复发率(3.3% *vs* 4.7%, *P*=0.579) For Masaoka-Koga pStage Ⅰ-Ⅱ tumors, the recurrence rate (3.3% *vs* 4.7%, *P*=0.579) was similar between the two groups

## 3 讨论

传统以来，胸骨正中劈开手术被认为是胸腺恶性肿瘤的标准术式，同时侧胸切口有时也被用来治疗一些侵犯胸壁的病例^[6, 7]^。近年来，以胸腔镜为代表的微创外科技术被应用于胸腺恶性肿瘤治疗之中，并得到了飞速的发展^[8-10]^。在国内，越来越多的医生和患者均愿意选择采用胸腔镜手术，这从本数据库中所纳入的国内18家大中心来看亦是如此。

相比开放手术，胸腔镜胸腺切除术在理论上的优势在于前纵隔区域的显露更加清晰，同时也能方便地探查同侧的胸腔并完成全胸腺切除和周围脂肪的切除。既往的研究^[11, 12]^表明，胸腔镜手术是比较安全的。本研究中胸腔镜组全胸腺切除率明显高于开放手术组，并且之前发表的本系列研究亦报道，胸腔镜组的手术时间、术中出血量、住院时间均明显少于开放组，这与现有的文献的结果相一致^[13]^。

胸腔镜胸腺切除术相比开放手术后的长期生存率的差异目前尚未有足够的依据。有零星的胸腔镜胸腺瘤切除术后发生肿瘤胸膜腔播撒的病例报道^[14, 15]^。Pennathur等^[16]^纳入40例患者的对照研究中报道，在经过了36个月的随访期之后，胸腔镜组和开放组的复发率和总体生存率没有统计学差异。而在本研究中，胸腔镜组相比开放组的复发率明显更低。不过，由于胸腺瘤的生物学恶性程度较低，即使发生了复发的病例仍然有长期生存率的希望^[17]^，这也许可以解释本研究中两组虽然在无瘤生存率方面具有差异（92% *vs* 83%, *P*=0.011），但在总体生存率方面却没有统计学差异（92% *vs* 92%, *P*=0.15）。为了排除本研究中可能存在的选择偏倚，我们进一步比较了病理分期为早期的胸腺瘤的患者预后情况，结果显示两组在复发率（3.3% *vs* 4.7%, *P*= 0.579）和总体生存率方面（89.4% *vs* 96.7%, *P*=0.582）没有差异。这也再次验证了对于早期胸腺瘤，胸腔镜手术能够提供与开放手术相似的肿瘤学疗效。

与既往文献^[18-21]^相一致的是，Masaoka-Koga分期和WHO分型再次被证明是胸腺瘤独立的预后因素。本研究中显示Masaoka-Koga分期为Ⅲ期和Ⅳ期的患者相比早期的患者其预后差的风险明显增加，然而在Ⅰ期与Ⅱ期之间则没有显著的差异。而从外科角度，Masaoka-Koga Ⅰ期和Ⅱ期的病例足以被胸腔镜手术所根治。与此同时，多因素分析也显示手术方式的选择并不影响预后，这再次支持了胸腔镜手术对于早期胸腺瘤治疗的适用性。除此之外本研究还发现辅助治疗也影响长期的生存情况，不过早期恶性胸腺瘤在根治性切除术后行辅助治疗的作用仍然存在争议^[22]^，对此需要今后的研究来明确。

本研究是迄今为止最大样本量的报道，仍然存在不足之处。由于手术方法是非随机的，不可避免地导致选择偏倚；此外回顾性研究的本质也无法避免内在的其他偏倚。因此今后需要进行倾向配比分析研究或者前瞻性的研究。另外，由于胸腺瘤的肿瘤学恶性程度不高，因此需要比其它肿瘤更长的随访时间来反映其长期的生存情况，或许十年生存率会是更好的评价指标。

## 4 结论

胸腔镜胸腺切除术是治疗早期胸腺恶性肿瘤安全有效的方法，与开放手术相比其围手术期效果更好、肿瘤学疗效一致。
